# Mental health and officiating performance

**DOI:** 10.3389/fpsyg.2025.1720108

**Published:** 2026-01-06

**Authors:** Nate Taylor, David Hancock, Philip Sullivan

**Affiliations:** 1 Department of Kinesiology, Brock University, St. Catharine’s, ON, Canada; 2 School of Human Kinetics & Recreation, Memorial University of Newfoundland, St. John's, NL, Canada

**Keywords:** mental health, performance, wellbeing, psychological distress, sport officials

## Abstract

**Introduction:**

Mental health challenges have been recognized as factors that can influence sport officials’ ability to perform effectively. For example, 90% of soccer officials believed common mental disorders could negatively affect refereeing performance. Therefore, this study aimed to uncover if mental health affects officiating performance.

**Methods:**

This study used a subjective rating of officiating performance (e.g., positioning, rule application, communication, etc.) and measures of mental health outcomes using the Kessler K10 psychological distress scale and the Warwick-Edinburgh mental wellbeing scale to determine the relationship between mental health and performance. A sample of 274 referees (85% male, 84% Caucasian, *M*_age_ = 47.3, *M*_experience_ = 19.0) from Canada Basketball participated in this study.

**Results:**

A Hierarchical Linear Regression predicting performance from psychological distress and wellbeing, after controlling for experience, was significant [*F*(3, 235) = 22.60, *p* < 0.001], accounting for 19% of the variation in officials’ performance.

**Discussion:**

This study demonstrates that the wellbeing of sport officials is a significant predictor of perceived performance, whereas psychological distress does not appear to directly influence performance.

## Introduction

Sports officials (i.e., umpires, judges, referees, and time/scorekeepers) play a crucial role in ensuring fair play, enforcing rules, and managing the dynamics of sport competitions (Webb et al., 2025). On top of the already demanding role that involves physical movement (in many sports), making quick/accurate decisions, and negotiating communication with athletes and coaches, officials’ encounter added stressors, most notably instances of abuse that can impact their mental health (Brick et al., 2022; Downward et al., 2023). The World Health Organization defines mental health as “a state of wellbeing in which the individual realizes his or her own abilities, can cope with the normal stresses of life, can work productively and fruitfully, and is able to contribute to his or her community” (World Health Organization, 2004, p. 13). Based on this definition, maintaining strong mental health is essential for officials to withstand the inherent stresses of their role and carry out their duties with confidence and effectiveness.

Recent research has highlighted the extremely high prevalence rates of verbal (e.g., intimidation, threats of harm, swearing, coercion, harassment, or humiliation) and physical (e.g., assault, hitting, punching, slapping, kicking, pushing, head-butting, hair-pulling, or biting) abuse experienced by sport officials and its detrimental impact on their mental health and willingness to continue officiating. Over 94% of Gaelic Games officials experienced verbal abuse, and 23% experienced physical abuse throughout their career, rates comparable to or exceeding those in other team sports (e.g., soccer, rugby; Brick et al., 2022). Importantly, abuse was associated with increased psychological distress, lower mental wellbeing, heightened anxiety and depressive symptoms, and greater intentions to quit officiating (Brick et al., 2022).

Further research has shown consistently high rates of abuse, with nearly half of rugby union and cricket officials reporting being abused at least twice each season and over 50% perceiving that such abuse is increasing, alongside concerns about insufficient training and organizational support (Webb et al., 2019). Similar patterns emerge from France and the Netherlands showing frequent verbal abuse and notable incidents of physical abuse, suggesting an entrenched culture of hostility toward officials in both countries (Webb et al., 2020). Moreover, research examining gendered experiences has revealed particularly toxic and male-dominated environments for female match officials, characterized by sexist and derogatory language, limited access to mental health support, and reports of poor mental health during and after matches (Webb et al., 2021). Together, these findings underscore that abuse of officials is a widespread and systemic issue that extends beyond specific sports or regions, reinforcing the need to examine how such environments shape officials’ mental health and performance.

Compounding the issues regarding wellbeing discussed above is the fact that sport officials tend to have lower levels of mental health literacy compared to athletes and coaches, which may hinder their ability to recognize and address these emerging problems effectively (Gorczynski and Thelwell, 2022). This discrepancy may stem from a lack of resources specifically designed to educate officials on critical topics such as mental health, wellbeing, and effective coping strategies. Unlike athletes and coaches, the more isolated nature of the role officials have (i.e., limited interactions with peers and infrequent opportunities to build relationships with other officials) may result in fewer opportunities to engage in discussions that promote an awareness and understanding of mental health, leaving a gap in their ability to recognize and address these issues.

Additionally, the officiating community faces a notable lack of social support, which has been identified as the second most common factor associated with mental health disorders within this group (Kilic et al., 2018). The isolated nature of officiating, characterized by limited interactions with peers and infrequent opportunities to build relationships with other officials, contributes to this lack of support. These conditions can leave officials feeling disconnected and without a network of peers to turn to when faced with mental health challenges. If not managed properly these sources of stress can develop into more serious mental disorders. For example, Gouttebarge et al. (2017) found that over the course of a season, new symptoms of common mental disorders emerged among professional football referees at rates of 10% for distress, 16% for anxiety or depression, 14% for sleep problems, 29% for eating disorder symptoms, and 8% for problematic alcohol use. Interestingly, other roles within sport have acknowledged the importance of social support for their mental health. For example, Pilkington et al. (2022) found that high performance coaches and support staff report similar prevalences of mental health issues to officials. Dissatisfaction with social support was the strongest predictor of psychological distress in the coaches and support staff, who work within a team-based context much more so than do officials. Importantly, Gouttebarge et al. found that 90% of soccer officials acknowledge that these common mental disorders can have a negative effect on their refereeing performance, underscoring a critical link between wellbeing and professional effectiveness.

While the effect of stress on sport officials’ mental health has been well documented, the direct implications this has for officiating performance remain less clear. Existing research has linked mental health concerns such as depression, anxiety, and stress to self-perceived performance difficulties (e.g., feeling a need to train harder for future contests or feeling useless as a referee; Lima et al., 2023). Furthermore, research examining the psychological demands of officiating further supports the need to investigate how mental health influences officials perceived performance. For example, Sors et al. (2019) showed that anxiety can meaningfully impair decision-making accuracy among basketball referees, particularly in high-pressure environments. Although anxiety alone did not produce a main effect, the authors found a significant interaction between anxiety level and crowd context: officials with high anxiety demonstrated reduced decision-making accuracy when exposed to a hostile crowd, whereas low-anxiety officials showed no decline. These findings indicate that anxiety heightens sensitivity to evaluative pressure, such as fears of being perceived as biased or making an incorrect call, which can disrupt cognitive processing under stress. Collectively, this literature highlights the importance of understanding how mental health shapes officials’ performance perceptions, especially within environments characterized by scrutiny, noise, and public evaluation.

Addressing this gap is essential for both safeguarding officials’ wellbeing and preserving the integrity of sport competition. Accordingly, the present study employs a correlational design to investigate relationships among psychological distress, wellbeing, and self-reported performance in sport officials. Specifically, it examines whether indicators of distress and wellbeing are associated with perceived officiating effectiveness. By clarifying how different aspects of mental health relate to performance outcomes, the findings aim to provide empirical evidence to help officiating organizations understand the implications of psychological wellbeing for performance. Ultimately, this knowledge can inform targeted interventions and policies that support both the mental health and professional effectiveness of sport officials.

## Methods

### Participants

Participants were recruited through Basketball Canada via email in March 2024. The final sample consisted of 274 basketball referees, all of whom were over the age of 18 and had fully completed the online survey. The sample was predominantly male (*n* = 233; 85%) and largely identified as Caucasian (84%), with an average age of 47.3 years (*SD* = 15.19). Participants had an average of 19.0 years of officiating experience (*SD* = 13.22), highlighting a thoroughly experienced population of officials with exposure to the demands of refereeing. The participants in this study represented a broad spectrum of officiating levels, including recreational (*n* = 9), competitive (*n* = 175), university/college (*n* = 60), national (*n* = 21), international (*n* = 8), and professional competition (*n* = 9).

### Procedure

Ethical clearance was granted by the PI’s institution’s REB in February 2024, ensuring that all research procedures adhered to ethical standards. Data collection was facilitated through an online survey that was designed using Qualtrics. The information and consent form were included in the Qualtrics survey.

Canada Basketball was contacted and agreed to assist with participant recruitment by distributing the survey to their accredited referees. A nationwide email invitation was sent to 4,189 officials registered with Canada Basketball (does not encompass all basketball referees in Canada), of whom 274 referees (6.5%) fully completed the survey. Surveys were completed in the second half of the season. While the response rate was modest, the final sample provided a broad representation of basketball referees in Canada from various officiating levels and regions. A sample of at least 251 can be considered representative of a population of 4,000, with a 90% confidence interval and 6% margin of error (Qualtrics, 2025). Furthermore, an *a priori* sample size calculation using G Power (Faul et al., 2007) for a hierarchical linear regression with three predictors with a medium effect size, power of 0.95 and alpha of 0.05 determined a minimal sample size of 107.

### Instruments

The survey included a brief demographic section. Participants were asked to report their age, sex, and race before completing the remaining measures. These variables were collected to describe the sample and ensure the methodological clarity required for potential replication.

#### Officiating performance

Officiating performance was assessed using an 8-item questionnaire designed to evaluate key aspects of refereeing proficiency. Participants were asked to self-rate their performance across eight dimensions: positioning, application of rules, game management, judgment, psychological skills (e.g., ability to remain calm and focused during pressure situations), presence, effort, and teamwork. These dimensions collectively represent key elements required for effective officiating.

Participants provided ratings based on their perceived performance during the past 30 days, using a Likert-type scale ranging from 1 (poor) to 9 (excellent). The responses were averaged to generate a self-rated officiating performance score, providing a measure of their recent performance levels. This measure, developed and validated by Hancock et al. (2024), was specifically designed for use in referee populations and has been demonstrated to be both reliable and valid for assessing officiating performance.

#### The Warwick-Edinburgh mental wellbeing scale (WEMWBS)

The Warwick-Edinburgh Mental Wellbeing Scale (WEMWBS) is a widely used and validated tool designed to measure mental wellbeing across diverse populations (Stewart-Brown et al., 2011; Tennant et al., 2007). The scale evaluates three core components of wellbeing: positive affect (e.g., feelings of happiness and optimism), satisfying interpersonal relationships, and positive functioning (e.g., sense of purpose and engagement in life). The WEMWBS has been extensively validated and is recognized for its strong psychometric properties. Initial validation work showed good content validity and a unidimensional factor structure, supported by confirmatory factor analysis (GFI = 0.93, AGFI = 0.80, RMSEA = 0.055; Stewart-Brown et al., 2011). The scale exhibits high internal consistency (Cronbach’s α = 0.89), with item–total correlations ranging from 0.52 to 0.80 and no evidence of floor or ceiling effects. Test–retest reliability is also strong (r = 0.83), indicating stability over time.

The scale comprises a series of statements and participants are asked to rate how well each statement reflected their experiences over the past 2 weeks (e.g., I’ve been dealing with problems well, and I’ve been thinking clearly). Responses were recorded on a 5-point Likert scale, ranging from 1 (none of the time) to 5 (all of the time). The scores from all items were summed to provide an overall measure of mental wellbeing, with higher scores indicating better mental wellbeing. The WEMWBS is particularly appropriate in this study because the measure is designed to assess general mental wellbeing across everyday life contexts. This aligns with our aim to examine officials’ overall mental wellbeing and how it relates to their perceptions of officiating performance, rather than evaluating wellbeing during officiating itself.

#### Kessler psychological distress scale (K10)

The K10 specifically evaluates the frequency and intensity of symptoms related to anxiety and depression that individuals have experienced over the most recent four-week period (e.g., during the last 30 days, about how often did you feel so sad that nothing could cheer you up?). The Kessler Psychological Distress Scale (K10) has demonstrated strong psychometric properties across large population studies. The scale shows good measurement precision, particularly in the upper range of psychological distress, with standard errors of standardized scores typically between 0.20 and 0.25 (Kessler et al., 2002). Its validity is well established: the K10 reliably discriminates between community cases and non-cases of DSM-IV psychiatric disorders, yielding areas under the ROC curve of 0.87–0.88 for disorders with Global Assessment of Functioning (GAF) scores of 0–70, and 0.95–0.96 for more severe disorders (GAF 0–50). These results indicate excellent sensitivity and specificity.

This scale consists of 10 questions, each addressing a specific emotional state, such as feeling nervous, hopeless, restless, or depressed. Participants responded to each item using a 5-point Likert scale, ranging from 1 (none of the time) to 5 (all of the time). The scores were summed to generate an overall distress score, with higher scores indicating greater levels of psychological distress. This tool is particularly valuable for identifying those who may be at risk of clinically significant mental health challenges. Furthermore, the K10 assesses symptoms of anxiety and depression experienced over the previous 4 weeks, which aligns closely with the 30-day timeframe used in the Officiating Performance measure. This recall timeline strengthens the conceptual fit between the two instruments and supports the appropriateness of using the K10 to examine how recent psychological distress relates to perceived officiating performance.

### Design

This study employed a correlational and cross-sectional design to explore the relationships between variables at a single time point. The research question was analyzed with a hierarchical multiple linear regression predicting self-reported performance from psychological distress (i.e, K10) and wellbeing (i.e., WEMBS) scores. Because experience has been found to be positively related to wellbeing and negatively related to distress (Carson et al., 2020; Lima et al., 2023) and positively related to performance (Lima et al., 2023; Neil et al., 2013), experience was entered in the first model and K10 and WEMWBS were entered, simultaneously, in the second model. Age and experience were highly correlated (*r* = 0.67, *p* < 0.001) in the present sample, therefore we chose to only include experience, not age as a variable in the model. All analyses were conducted with SPSS 29.0.1.

## Results

There was some missing data within the three variables for analyses. Specifically, 2.4% of the data was missing, and was found to be Missing Completely at Random [*χ* (8) = 4.68, *p* = 0.79]. These values were replaced with the mean of nearby places. This approach estimates missing values based on the pattern of surrounding data and allows for a reasonable prediction of continuous variables while allowing a greater degree of variation in data than using the series mean would. Prior to analyses, data was checked for assumptions of MLR, including normality, homoscedasticity, and linearity. Examination of Q: Q plots and boxplots suggested abnormal distribution, particularly skewness, due to outliers. A total of 31 cases identified through boxplots as outliers were removed. This resulted in a sample size of 241 for analysis. At this point visual analyses of all three variables suggested that assumption of normality was upheld. Scatterplots of all pairs of variables showed that the assumptions of linearity and homoscedasticity were also upheld, and Cook’s distances showed that no cases were multivariate outliers (i.e., all values < 0.05). VIF (1.68–1.69) and Tolerance (5.91–5.94) statistics indicated an absence of multicollinearity between K10 and WEMWBS. Table 1 gives the descriptive statistics and Cronbach’s alphas of the performance score, K-10, and WEMWBS. All variables showed acceptable levels of internal consistency (i.e., α > 0.70).

**Table 1:** Descriptive statistics for all variables.

Factor	M (SD)	α
Performance	7.87 (0.64)	0.83
K10	16.60 (5.05)	0.88
WEMWBS	53.51 (7.33)	0.90

Performance scores range from 1 to 9, K10 ranges from 10 to 50, WEMWBS ranges from 14 to 70. Higher scores in all cases indicate more of that attribute.

The first step of the Multiple Linear Regression, with experience as the sole predictor of performance, was significant [*F*(1, 237) = 8.61, *p* < 0.05]. The second model, which added the predictors of K10 and WEMWBS scores was significant [*F*(3, 235) = 22.60, *p* < 0.001; *ΔR^2^* = 0.19, *p* < 0.001]. This model accounted for 22% of the variation in officials’ performance. Both the amount of variation in the final model and he change from model 1 to 2 constitute a moderate effect. Wellbeing was a significant predictor of performance however psychological distress was not. Table 2 summarizes the regression model.

**Table 2:** Hierarchical multiple regression for officiating performance.

Variable	95% CI for B		β	R^2^	ΔR^2^
	LL	UL			
Step 1				0.04^*^	
Constant	7.59	7.85			
Experience	0.003	0.015	0.19^***^		
Step 2				0.22^***^	0.19^***^
Constant	4.75	6.57			
Experience	0.004	0.014	0.20^***^		
K10	−0.016	0.020	0.02		
WEMWBS	0.025	0.050	0.45^***^		

## Discussion

The results of this study demonstrate that the wellbeing of referees is highly associated with their perceptions of performance. In short, referees who reported higher levels of wellbeing believed they perform better in their roles. The significant relationship between wellbeing and officiating performance highlights the importance of fostering a positive state of mental health among referees to facilitate optimal performance. In contrast, psychological distress did not significantly predict performance. This result suggests that while psychological distress may impact other areas of life, it does not appear to have a direct or measurable influence on refereeing performance in this context. One plausible explanation is the likelihood of underreporting psychological distress among officials. Given the performance-driven and evaluative nature of officiating, individuals may minimize or downplay distress due to stigma, fear of appearing incapable, or cultural norms within sport that prioritize toughness and composure (Schwenk, 2000). As a result, self-reported distress levels may not accurately reflect their true psychological state, reducing the likelihood of detecting a significant relationship.

Another explanation is that officials experiencing psychological distress may still be capable of performing their duties effectively. As described in Keyes (2002) dual-continuum model, mental illness and mental well-being are related but distinct constructs, meaning an individual can experience symptoms of distress while still maintaining behavioral functioning, and task execution. Thus, distress may influence their overall well-being without necessarily impairing the measurable aspects of their refereeing performance. After controlling for experience (19 years), wellbeing remained a significant predictor of performance. This demonstrates that psychological attributes such as wellbeing contribute uniquely to referees’ effectiveness, beyond the influence of demographic factors. Although prior research suggests that older and more experienced referees tend to demonstrate stronger coping skills, experience fewer negative emotional symptoms, and are less vulnerability to mental health challenges (Carson et al., 2020; Lima et al., 2023; Neil et al., 2013; Slack et al., 2013), the present findings indicate that wellbeing itself, rather than experience, was the more relevant predictor of officiating performance. This suggests that while demographic factors may provide opportunities to develop resilience, it is the referees’ current state of psychological wellbeing that ultimately drives effectiveness. In other words, experience may help referees learn to cope, but without sufficient wellbeing, performance is still compromised.

The significant relationship between WEMWBS and officials’ performance is consistent with the notion that wellbeing enables effective functioning and the ability to cope with stress (World Health Organization, 2004). This aligns with existing research underscoring the importance of effective coping strategies to maintain decision accuracy under pressure (Neil et al., 2013). Although psychological distress is well documented among sport officials, with general incidence rates of approximately 10% (Gouttebarge et al., 2017), it did not significantly predict performance in the current study. On average the K10 scores with the current sample were relatively low; the mean score fell into the range of “likely to be well” per typical scoring. It may be in this sample that the limited association between distress and performance may be due to this highly functional level of distress. While psychological distress has been linked to burnout and attrition among officials (Brick et al., 2022; Downward et al., 2023), its immediate impact on performance may be less pronounced when officials possess strong resilience and regulatory skills. This apparent disconnect between wellbeing and distress as predictors of performance highlights the need for a broader conceptual framework to interpret these findings.

The Dual Continuum Model of Mental Health offers such a lens. Unlike traditional views that assume mental illness and mental health exist at opposite ends of the same continuum, the Dual Continuum model conceptualizes them as two interconnected but distinct constructs (Keyes, 2002). Within this model, an individual may be both high in mental health (e.g., flourishing socially and emotionally) while still experiencing mental illness (e.g., having a diagnosis of an eating disorder). Alternatively, just because someone is not experiencing a mental illness does not mean they are mentally healthy—they may be languishing socially or emotionally. In the current study, wellbeing, measured via the WEMWBS and psychological distress, measured by the K10, were operational definitions of mental health and mental illness, respectively. Both of these are commonly used in this way (Iasiello et al., 2020).

One of the implications of the Dual Continual Model is that if mental health and mental illness are separate (albeit related) constructs, then they may show different relationships to the same outcome. For example, outcomes like curiosity (Jovanovic and Brdaric, 2012) and problem solving (Karademas, 2007) have been found to be positively related to wellbeing but related negatively or not at all to mental illness. In the present study, we see that the K10 was unrelated to the outcome of performance, while WEMWBS scores were significantly related. Not only is this finding consistent with the Dual Continuum Model, but similar outcomes have been found in other fields. For example, it has been found that wellbeing, but not mental illness, had positive relationship with academic attitudes (Suldo et al., 2011) and performance (Lyons et al., 2013) as well as employment (Huppert and Whittington, 2003).

Importantly, these results move beyond simply identifying distress as a barrier. Promoting flourishing and positive mental states should therefore be a core focus of sport organizations. For officiating, this means that interventions designed solely to reduce stress or manage burnout may be insufficient. Therefore, initiatives that focus on actively cultivating wellbeing (e.g., peer support, resilience training, positive psychology strategies, and organizational culture change) may provide a more effective means of sustaining performance. Taken together, the Dual Continuum Model not only provides a valuable theoretical framework for interpreting the results of this study but also points toward a shift in applied practice. Fostering wellbeing is not just about protecting officials from harm, but about enabling them to thrive in a demanding environment.

One particularly promising approach to fostering wellbeing is targeting mental health literacy, as higher literacy can enhance knowledge, strengthen help-seeking attitudes, reduce stigma, and promote sustained engagement with mental health resources, all of which contribute to officials’ overall wellbeing (Bu et al., 2020). This is especially relevant for officials, as research shows that soccer referees report comparatively low literacy levels, scoring below athletes and athletic staff on the same measures (Gorczynski and Thelwell, 2022; Sullivan et al., 2018). These challenges are compounded by a sporting culture that often frames mental health concerns as weakness, reinforcing barriers to support-seeking (Lundqvist and Andersson, 2021). Strengthening mental health literacy in this context is therefore critical not only for protecting officials’ wellbeing but also for sustaining their performance under pressure.

Given the clear links between wellbeing and performance found in this study, governing bodies hold both an ethical and performance-related responsibility to implement proactive policies that prioritize mental health. Such measures could include zero-tolerance policies on abuse, accessible counselling services, and the integration of mental health education into referee development pathways. These initiatives not only safeguard officials’ wellbeing but also address retention challenges, as chronic psychological strain and abuse remain leading causes of referee attrition (Brick et al., 2022). However, we must consider the practicality of such policies. Acknowledging that zero-tolerance policy for abuse against officials may not be feasible, other measures should be examined that may help officials. Interventions to build resiliency (Vella et al., 2021), and interpretation of anxiety or mindfulness (Bühlmayer et al., 2017) in particular may be quite valuable.

It is important to note that the predominance of male officials in our sample reflects the broader gender distribution within officiating. This pattern is consistent with other large-scale studies of sports officials, which similarly report a strong overrepresentation of men. For example, Carrington et al. (2025) found that of 402 officials surveyed during instrument development, 349 were male and only 50 were female. As such, our sample closely mirrors the demographic structure of the officiating population, supporting the representativeness of our findings.

In this study, data was collected solely from basketball officials, which restricts the generalizability of the findings to other sport officials who may face different stressors, organizational cultures, and performance demands. Additionally, data collection occurred at one timepoint in the season. Prior research on sport officials has shown that symptoms of common mental disorders fluctuate across the season, with one-season incidence rates reported at 10% for distress, 16% for anxiety/depression, 14% for sleep disturbances, 29% for eating disorders, and 8% for alcohol-related problems (Gouttebarge et al., 2017). These fluctuations suggest that the current findings may not fully capture variations in mental health and performance across an entire season. Longitudinal studies tracking officials throughout a season would provide a more comprehensive understanding of these dynamics.

The study included referees from a range of levels, however, the sample was strongly gender-imbalanced, with approximately 85% identifying as male. This reflects the broader gender distribution within basketball officiating in Canada, but it nonetheless limits the ability to examine potential gender-based differences in mental health experiences. Prior research suggests that gender may shape how officials encounter stress, abuse, and support resulting in differences in how mental health is impacted (Tingle et al., 2022; Webb et al., 2021). As such, the findings may not fully capture the perspectives of women or gender-diverse officials, and future research should aim to recruit a more gender-balanced sample to explore these differences more explicitly.

One notable limitation lies within removing 11% of the observations which were necessary to meet statistical assumptions and ensure the validity of the analyses, this decision resulted in a notable reduction in the sample size. Excluding this proportion of cases may have influenced the stability and generalizability of the findings, particularly given the variability common in social science data. As such, the results should be interpreted with caution, acknowledging that the excluded data could have contributed additional nuance to the observed patterns. Additionally, the different reference periods of the measures used. The WEMWBS assesses wellbeing over the past 14 days, the K10 measures psychological distress over the past 4 weeks, and the officiating performance questionnaire covers the previous 30 days. While the 4-week and 30-day periods are roughly equivalent, wellbeing may fluctuate more rapidly than distress or performance, which could affect the observed relationships in the regression model. Therefore, temporal differences between the measures should be considered when interpreting the findings.

To build on the present findings, future research should explore the mechanisms through which wellbeing influences officiating performance. In particular, qualitative factors including sense of purpose, social support, and professional identity, could uncover insights that quantitative measures alone may overlook. A deeper understanding of how officials interpret and make meaning of their mental health experiences in relation to performance would inform tailored interventions and enhance theoretical frameworks linking mental health and performance. Such research could guide the development of targeted strategies that reflect the unique challenges and demands faced by officials while supporting their wellbeing and professional effectiveness. Furthermore, the sample included referees from multiple officiating levels, it is possible that mental health experiences and performance-related outcomes differ across these groups. The present study was not designed to compare these subgroups. However, future research should examine level-specific differences to better understand how competitive context and officiating demands shape referees’ mental health and subsequently their performance.

In conclusion, this study demonstrates that the wellbeing of sport officials is a significant predictor of perceived performance, whereas psychological distress does not appear to directly influence performance. These findings underscore the critical role of positive mental health resources in supporting officials’ effectiveness, highlighting that fostering wellbeing may be more impactful than relying solely on demographic or experiential factors. Importantly, the results emphasize the need for a holistic approach to mental health promotion that combines psychological skills training (e.g., emotional regulation, resilience, mental toughness) with organizational initiatives aimed at reducing stigma, enhancing mental health literacy, and providing accessible support. Future research using longitudinal, experimental, and qualitative designs is essential to clarify causal relationships, uncover underlying mechanisms, and guide targeted interventions tailored to the unique demands of officiating. By prioritizing officials’ psychological wellbeing, governing bodies can not only enhance performance but also protect against attrition and support a sustainable and high-functioning officiating community.

## Data Availability

The data supporting the findings of this study are restricted due to ethical considerations and participant confidentiality associated with mental health survey data. Data may be made available from the corresponding author upon reasonable request, subject to institutional ethics approval.
